# A first draft genome of holm oak (*Quercus ilex* subsp. *ballota*), the most representative species of the Mediterranean forest and the Spanish agrosylvopastoral ecosystem “*dehesa*”

**DOI:** 10.3389/fmolb.2023.1242943

**Published:** 2023-10-12

**Authors:** María-Dolores Rey, Mónica Labella-Ortega, Víctor M. Guerrero-Sánchez, Rômulo Carleial, María Ángeles Castillejo, Valentino Ruggieri, Jesús V. Jorrín-Novo

**Affiliations:** ^1^ Agroforestry and Plant Biochemistry, Proteomics and Systems Biology, Department of Biochemistry and Molecular Biology, University of Cordoba, UCO-CeiA3, Cordoba, Spain; ^2^ Royal Botanic Gardens, Kew, Richmond, United Kingdom; ^3^ Biomeets Consulting ITNIG—Carrer d’ Alaba 61 08005 Catalonia, Barcelona, Spain

**Keywords:** *Quercus*, *Quercus ilex*, long-read assembly, PacBio HiFi sequencing, decline syndrome

## Abstract

The holm oak (*Quercus ilex* subsp. *ballota*) is the most representative species of the Mediterranean Basin and the agrosylvopastoral Spanish “*dehesa*” ecosystem. Being part of our life, culture, and subsistence since ancient times, it has significant environmental and economic importance. More recently, there has been a renewed interest in using the *Q. ilex* acorn as a functional food due to its nutritional and nutraceutical properties. However, the holm oak and its related ecosystems are threatened by different factors, with oak decline syndrome and climate change being the most worrying in the short and medium term. Breeding programs informed by the selection of elite genotypes seem to be the most plausible biotechnological solution to rescue populations under threat. To achieve this and other downstream analyses, we need a high-quality and well-annotated *Q. ilex* reference genome. Here, we introduce the first draft genome assembly of *Q. ilex* using long-read sequencing (PacBio). The assembled nuclear haploid genome had 530 contigs totaling 842.2 Mbp (N50 = 3.3 Mbp), of which 448.7 Mb (53%) were repetitive sequences. We annotated 39,443 protein-coding genes of which 94.80% were complete and single-copy genes. Phylogenetic analyses showed no evidence of a recent whole-genome duplication, and high synteny of the 12 chromosomes between *Q. ilex* and *Quercus lobata* and between *Q. ilex* and *Quercus robur*. The chloroplast genome size was 142.3 Kbp with 149 protein-coding genes successfully annotated. This first draft should allow for the validation of omics data as well as the identification and functional annotation of genes related to phenotypes of interest such as those associated with resilience against oak decline syndrome and climate change and higher acorn productivity and nutraceutical value.

## Introduction

The holm oak (*Quercus ilex* subsp. *ballota*) is included in the 640 recorded species in the “Plants of the World Online” database within the genus *Quercus* (de Rigo and Caudullo, 2016). Oaks is one of the most important clades of woody angiosperms in the Northern Hemisphere in terms of species diversity, ecological dominance, and economic value (Backs and Ashley, 2021). It has been part of life, culture, and subsistence since ancient times (Leroy et al., 2020). Moreover, the holm oak is a priority species for afforestation in semi-arid regions due to its high drought tolerance (Barbeta and Peñuelas, 2016) and its phenotypic plasticity in response to varying edaphic conditions (Laureano et al., 2016).

The holm oak is the dominant species in the Mediterranean forest and agrosylvopastoral ecosystem “*dehesa*,” with relevance from an environmental and economic point of view, especially in rural areas (Plieninger et al., 2021). The most remarkable economic benefit is related to its fruit, the acorn, used for feeding black Iberian pigs (*Sus scrofa domesticus* L.) (Diaz-Caro et al., 2019). Recently, there has been a renewed interest in using *Quercus* spp. as a source of functional food due to the increasing demand for alternative plant species, including forest trees, for dietary diversification, and for sustainable food production (Vinha et al., 2016; Lopez Hidalgo et al., 2021).

Currently, *Q. ilex* is seriously threatened by several factors including aging tree populations, overexploitation coupled with poor regeneration capacity, inappropriate livestock management, and biotic (e.g., *Phytophthora cinnamomi*) and abiotic stresses (e.g., extreme temperatures and extended drought periods). Together, these factors likely contribute to the so-called holm oak decline syndrome, which has increased tree mortality and seriously damaged the *dehesa* (Brasier, 1992; Camilo-Alves et al., 2013; Ruiz-Gómez et al., 2018; San-Eufrasio et al., 2021). The main priority for *Q. ilex* conservation and sustainable exploitation is the establishment of breeding programs aimed at increasing acorn production and resilience to the decline syndrome (Cortés et al., 2020). These programs will contribute to the proper and sustainable management, conservation, and exploitation of *Q. ilex* and related ecosystems (Plomión et al., 2016). A better understanding of this species at the biochemistry, molecular biology, and biotechnology levels will facilitate these breeding programs, as it has been the case with many herbaceous and other woody species (Muthuramalingam et al., 2022). As with many long-lived, non-domesticated, and allogamous species, the most plausible breeding strategy for *Q. ilex* is the use of genome-wide association studies (GWAS) to identify elite genotypes associated with phenotypes of interest (e.g., high acorn productivity, high content in phenolic compounds, and resilience to adverse biotic and abiotic stresses; Maldonado-Alconada et al., 2022).

To date, the number of publications focusing on the molecular aspects of *Q. ilex* biology has been limited due to its challenging biological characteristics (long life cycle, allogamy, and high phenotypic variability) and its recalcitrance as an experimental system (Maldonado-Alconada et al., 2022). However, despite these difficulties, our group has, to some extent, characterized the genetic structure and diversity of *Q. ilex* using DNA markers, microsatellites, transcriptomics, proteomics, and metabolomics (Maldonado-Alconada et al., 2022). These different approaches have allowed us to study seed germination, responses to biotic and abiotic stresses and their interaction, and the integration between molecular and morphometric data (Maldonado-Alconada et al., 2022). However, our research has so far been constrained by the lack of a *Q. ilex* reference genome, so the picture provided by these previous studies is still incomplete. The sequencing of the *Q. ilex* genome will add to the list of many recently published *Quercus* reference genomes (Plomión et al., 2016; Sork et al., 2016; Plomión et al., 2018; Ramos et al., 2018; Ai et al., 2022; Han et al., 2022; Liu et al., 2022; Sork et al., 2022; Zhou et al., 2022; Wang et al., 2023) and will allow us to interpret *Q. ilex* omics data in terms of its own gene products rather than relying on orthologs from other *Quercus* species. This should open new research venues related to the genetic makeup of this species, the identification of novel genes, allelic variants, and epigenetic marks, and will allow for studies on broader topics such as phylogenetics, hybridization and introgression patterns, and breeding programs informed by GWAS (Plomión et al., 2018; Stocks et al., 2019; Sork et al., 2022).

Many recently reported *de novo* genome assemblies have been carried out using next-generation short-read sequencing technologies such as Illumina or 454 platforms (Xia et al., 2017; Zhao et al., 2017), which tend to produce very fragmented genomes. Pacific BioSciences (PacBio) has developed a third-generation sequencing technology to assemble large and complex plant genomes producing tens of thousands of long individual reads (up to ∼40 kb) (Roberts et al., 2013; Paajanen et al., 2019). Here, we generate a draft genome assembly for *Q. ilex* (NCBI’s BioProject repository, ID: PRJNA687489) using the single-molecule real-time (SMRT) sequencing technology from PacBio. We reveal genomic features of *Q. ilex*, including nuclear and chloroplast sequences, repetitive sequences, and gene annotations. We also perform ortholog identifications and comparative genomics analyses to investigate possible gene duplication events, gene family expansions and contractions, and the degree of collinearity between *Q. ilex* chromosomes and other *Quercus* species. This first draft should allow us to validate the omics data previously obtained by our research group (Maldonado-Alconada et al., 2022) and facilitate the genetic enhancement of *Q. ilex* including the identification and functional annotation of genes associated with phenotypes of interest, such as resilience against oak decline syndrome and climate change or increased acorn productivity. Additionally, it will help us to understand the phylogenetic relationships among species of the genus *Quercus*.

## Materials and methods

### Genomic DNA extraction and PacBio sequencing

Leaves were collected from a mature, healthy *Q. ilex* tree located in Aldea de Cuenca, Fuente Obejuna, Córdoba, Andalusia, Spain (UTM 30S 276751 4245466 datum ETRS89), in November 2019 (Supplementary Figure S1). Upon arrival at the laboratory, leaves were washed with 2% sodium hypochlorite and abundantly rinsed with distilled water. For long-read sequencing, extraction of high-molecular-weight (HMW) genomic DNA from leaves was carried out using the cetyl trimethyl ammonium bromide (CTAB) method (Murray and Thomson, 1980) with slight modifications. In brief, 4 g of fresh leaves were macerated in liquid nitrogen, and 1 g of polyvinylpolypyrrolidone (PVPP) was added per each 20 mL of CTAB due to the high concentration of phenolic compounds in *Q. ilex* leaves. Once extracted, DNA was treated with RNAse (10 mg/mL, Thermo Fisher Scientific Inc., Waltham, MA, United States) for 1 h 30 min at 37°C, and the quality and concentration were determined by 1% agarose gel electrophoresis and using a Qubit version 3.0 Fluorometer (Thermo Fisher Scientific Inc., Waltham, MA, United States), respectively.

To determine whether the target individual was a pure *Q. ilex*, we compared its genotype with genotypes from other nine *Q. ilex* and 10 *Quercus suber* mature individuals using 20 microsatellite markers as previously described by Fernández i Marti et al. (2018). PCR amplifications were conducted in a T100™ Thermal Cycler (Bio-Rad, Hercules, CA, United States), and PCR products were analyzed on an ABI Prism 310 capillary electrophoresis system (Applied Biosystems, Foster City, CA, United States). Size alignment and quality control were analyzed using GeneMapper version 3.7 (Applied Biosystems, Foster City, CA, United States). We performed a principal component analysis (PCA) including all 20 microsatellite markers using the R package Adegenet (Jombart, 2008).

The target DNA sample was used to construct a PacBio SMRT sequencing library at the DNA Sequencing & Genotyping Center at the University of Delaware. Purified HMW DNA was fragmented to approximately 20 kb using Megaruptor version 3.0 (Diagenode, Denville, NJ, United States). Purified HMW DNA (10 μg) was used as a template to construct a SMRTbell HiFi library using Express Template Prep version 2.0 (Pacific Biosciences, Menlo Park, CA, United States) as per the manufacturer’s protocol. The quality of the HiFi library was assessed using the Qubit 3.0 Fluorometer (Thermo Fisher Scientific Inc., Waltham, MA, United States) and the Agilent Femto Pulse System (Agilent Technologies, Santa Clara, CA, United States). The HiFi library was run on two Sequel IIe system 8M SMRT cells using sequencing chemistry version 3.0 with 4 h pre-extension and 30 h movie time. Two runs of HiFi PacBio generated reads covering 64.3X of the genome assuming an *a priori* genome size of 850 Mbp. Although reads generated by HiFi PacBio are usually of high quality, an additional rough evaluation of nucleotide (A, C, G, and T) distribution was performed to eliminate any remaining low-quality reads. Reads showing a skewed nucleotide distribution (approximately 1%) were removed.

### *De novo Q. ilex* genome assembly

Filtered reads were used as input for a *de novo* assembly approach undertaken using NextDenovo version 2.5.0 software (key parameters: seed_depth = 45, nextgraph: -a1) (https://github.com/Nextomics/NextDenovo). The software was run in a “hifi” assembly mode, which skips the read correction step and produces a consensus of reads using a string graph algorithm. Contigs were separated into primary contigs and associated secondary contigs using the Purge_haplotigs pipeline version 1.1.1 (key parameter: -l 7, -m 45 and -h 180) (https://bitbucket.org/mroachawri/purge:haplotigs/src/master) (Roach et al., 2018) (Supplementary Figure S2). In regions with homologous contigs (e.g., heterozygous regions) in the haploid assembly, reads were split between the two contigs resulting in approximately half the read depth for those contigs. Based on the plot distribution, specific cut-offs were set to separate and flag low-quality contigs, secondary/alternative contigs, and high-coverage contigs (i.e., putative organelle genomes). Contigs, showing a very high coverage and forming a circular sequence, were blasted against the GenBank Non-Redundant Protein Sequence database (NCBI-BLAST) to verify matches with organelle genomes. The completeness of the genome assembly, generated by using primary contigs, was evaluated using Benchmarking Universal Single-Copy Orthologs (BUSCO) version 5.0 (key parameters: default, -l viridiplantae_odb10) (Simão et al., 2015). Using a chromosome-based genome assembly of a close and syntenic species (*Q. lobata*, valley oak NCBI ID = GCF_001633185.2, ValleyOak release 3.2, with RefSeq annotation when this work was performed), contigs were scaffolded at the chromosome level using RagTag (key parameters: scaffolding) (Alonge et al., 2019). Each *Q. ilex* contig was mapped against the chromosomes of the *Q. lobata* genome and scored for confidence according to grouping, location, and orientation.

### Repetitive sequences

We generated a high-quality non-redundant transposable element (TE) library for the *Q. ilex* genome using the Extensive *de novo* TE Annotator (EDTA) pipeline (https://github.com/oushujun/EDTA) (Ou et al., 2019). The pipeline combines a suite of best-performing packages (LTR_FINDER, LTR_harvest, LTR_retriever, Generic Repeat Finder, TIR-Learner, HelitronScanner, and RepeatMasker), and it was designed to filter out false discoveries in raw TE candidates and generate high-quality non-redundant TE libraries. The inbuilt package RepeatModeler version 2.0.1 (key parameters: default) (Bedell et al., 2000), which identifies any remaining TEs which might have been overlooked by the EDTA algorithm (--sensitive 1), was also used in the workflow. TE identification was performed using RepeatMasker version 1.332 (key parameters: -s -nolow -norna -nois -e rmblast -gff) (Bedell et al., 2000) with the NCBI/RMBLAST version 2.6.0+ search engine.

### Genome prediction and functional annotation

The annotation pipeline integrated different tasks starting from the fetching of raw data from public and/or private repositories (e.g., expressed sequence tags (ESTs), protein collections, RNA-Seq datasets, and other relevant sequences) to sequence alignment, prediction of gene models, and their functional annotations. We used MAKER2 (key parameters: default) (Campbell et al., 2014) as the core module for structural genome annotation. The software synthesizes a final annotation based on quality evidence values. Here, evidence was based on previously published transcripts, proteins, and data generated in this study as follows: 1) RNA-Seq datasets generated for *Q. ilex* by our research group (Guerrero-Sánchez et al., 2017; Guerrero-Sánchez et al., 2019; Guerrero-Sánchez et al., 2021) (NCBI accession codes: SRR11050903 and SRX2993508), 2) RNA-Seq datasets from *Q. ilex* seedling leaves under drought conditions generated by Madritsch et al. (2019) (NCBI accession codes: SRX4725055 and SRX4725058) using Augustus gene models produced by Braker2 (Brůna et al., 2021), 3) 22,000 reviewed proteins from UniProtKB (taxa rosids), 4) two sets of proteins from two closely related species (*Q. lobata* and *Juglans regia*) [NCBI accession codes: GCF_001633185.2 (*Q. lobata*) and GCA_001411555.2 (*J. regia*)], 5) *Quercus* EST collection [downloaded from the NCBI using “EST” and “*Quercus*” as keywords (download date: June 2022)], and 6) the curated TE library produced by the EDTA pipeline generated in this work. tRNAs were predicted using tRNAscan-SE (key parameter: default) (Lowe and Eddy, 1997). Tools from the AGAT v0.9.1 (https://github.com/NBISweden/AGAT) package were used to refine the annotation. The gene set annotation was checked to find cases where different gene features have coding sequences (CDS) that overlap and then merged (agat_sp_fix_overlapping_genes.pl), as well as the longest isoform was kept (agat_sp_keep_longest_isoform.pl).

To assign functional descriptions, Gene Ontology (GO) terms, and Kyoto Encyclopedia of Genes and Genomes (KEGG) pathway information to the gene models, sequences (transcripts/proteins) were functionally annotated with TRINOTATE version 2.0 (key parameters: protDB = uniprot_3745 (Viridiplantae), transdecoder + diamond + pfam) (https://github.com/Trinotate/Trinotate/wiki).

### Chloroplast genome assembly and annotation

Among all the contigs, one (ctg011470, 142.3 Kbp) matched chloroplast sequences in the NCBI (best match “*Medicago sativa* chloroplast, complete genome,” identity 99%). Chloroplast annotation was performed using the GeSeq tool (key parameters: default) (Tillich et al., 2017). GeSeq compares input sequences against reference databases using BLAT (Kent, 2002). We used additional tools (i.e., ARAGORN, tRNA-scan, and Chloë version 0.1.0) to annotate tRNAs, rRNAs, and CDS. Finally, OrganellarGenomeDRAW (Greiner et al., 2019) was used to convert annotations into a circular graphical map.

### Ortholog identification

OrthoFinder version 2.5.4 (Emms and Kelly, 2015; Emms and Kelly, 2019) (key parameters: default) was used to identify orthologous and duplicated nodes across *Q. ilex* and *Q. lobata* (ValleyOak3.0), *Q. suber* (CorkOak1.0), *Q. robur* (dhQueRobu3.1), *Populus trichocarpa* (Pop_tri_v3), *J. regia* (Walnut_2.0), and *Arabidopsis thaliana* (TAIR10). Protein sequences for each species were pre-processed to extract the longest transcript per gene using an OrthoFinder script (primary_transcript.py). Trees and plots were built using the Bioconductor library “cogeqc” (https://bioconductor.org/packages/release/bioc/vignettes/cogeqc/inst/doc/vignette_02_assessing_orthogroup_inference.html). The tree was an output of OrthoFinder (https://github.com/davidemms/OrthoFinder). Following the OrthoFinder manual, species tree is a STAG species tree inferred from all orthogroups, containing STAG support values at internal nodes and rooted using STRIDE.

### Genome synteny and whole-genome duplication analysis

Synteny analysis was performed between *Q. ilex* and *Q. lobata*, between *Q. ilex* and *Q. robur*, and between *Q. ilex* and *J. regia* using JCVI v1.3.3 (Tang et al., 2008) with the following parameters: -cscore = .99; -minspan = 30. Dot plots, synteny patterns, and macrosynteny visualizations were obtained using the graphics.dotplot, compara.synteny depth, and graphics.karyotype functions (https://gitbuh.com/tanghaibao/kcvi), respectively.

Whole-genome duplication (WGD) events were inferred across *Q. ilex* and two species of the genus *Quercus* (*Q. suber* and *Q. lobata*), with *J. regia* being used as the outgroup species. WGD analyses were performed using Ksrates v1.1.1 (key parameters: default) (Sensalari et al., 2022). The program compares paralog and ortholog Ks distributions derived from CDS and estimates differences in synonymous substitution rates across the included species. It then generates an adjusted mixed plot of paralog and ortholog Ks distributions. This allows us to assess the relative phylogenetic positioning of presumed WGD and speciation events.

### Gene family evolution analyses

In order to explore possible gene family expansions and contractions, we selected ortholog sequences from the seven species obtained through OrthoFinder (i.e., *Q. ilex*, *Q. suber*, *Q. lobata*, *Q. robur*, *J. regia*, *P. trichocarpa*, and *A. thaliana*). Since ancestral state reconstruction requires at least two species per clade of interest, we only used gene families with gene copies present in at least two species. In addition, orthogroups with more than 100 gene copies in one or more species were removed, since big gene families cause the variance of gene copy number to be too large and lead to non-informative parameter estimates. We produced a rooted phylogenetic tree using OrthoFinder, which was used as a probabilistic model to infer family expansions and contractions. The tree was made ultrametric using an OrthoFinder accessory script and setting 121 MYA as the total age of the tree in millions of years. This value represents the median adjusted time of divergence between *A. thaliana* and the *Quercus* clade calculated from the TimeTree5 database (Kumar et al., 2022). Gene family evolution analyses were performed using the Computational Analysis of gene Family Evolution (CAFE) algorithm (De Bie et al., 2006) with the lambda value set at 4.54495e-09. This parameter was calculated automatically by the software as the maximum likelihood value given the gene families in the data file. CAFE computes a “family-wide *p*-value” that was used to predict contracted/expanded families.

## Results and discussion

### *De novo Q. ilex* genome assembly

In the genus *Quercus*, both *Q. ilex* and *Q. suber* are sympatric in the Mediterranean area, and hybrids have been identified in natural populations (Burgarella et al., 2009; López De Heredia et al., 2018). *Q. ilex* and *Q. suber* individuals clustered at different sides of the PC1 axis, indicating an allopatric speciation of this individual (Supplementary Figure S3). We employed PacBio technologies to sequence the genome of *Q. ilex* subps. *ballota* [as defined by Flora Ibérica (Amaral, 1983), also known as *Quercus ilex* subsp. *rotundifolia* or *Quercus rotundifolia*], resulting in the generation of a diploid reference genome. First, we assembled 1,166 contigs totaling a genome size of 1,000,594,563 bp. The N50 and the average contig sizes were 2.63 and 0.85 Mbp, respectively (Supplementary Table S1). This initial genome assembly was larger than a previous estimate using flow cytometry (935.0 Mbp) (Rey et al., 2019). This difference can be explained by regions of very high heterozygosity leading to problems during genome assembly (Kajitani et al., 2014). For example, once a pair of allelic sequences exceeded a certain threshold of nucleotide diversity, these regions were assembled as separate contigs, rather than the expected single haplotype-fused contig, resulting in a significantly larger genome size. Moreover, the initial assembly included primary, secondary, and organelle (chloroplast and mitochondria) contigs. After these were separated using Purge_haplotigs (Roach et al., 2018), we obtained a total of 530 primary contigs (total size: 842.2 Mbp) and 616 secondary contigs (total size: 157.3 Mbp), and 20 (total size: 1.1 Mbp) were flagged as artifacts (i.e., too low or too high coverage). The final assembly of the nuclear haploid genome (842.2 Mbp) (Table 1) had a contig N50 size of 3.3 Mbp, indicating a *de novo* assembly with a high level of continuity. The size of the nuclear haploid genome is similar to that of other species of the genus *Quercus* (Table 1); however, the contig N50 of *Q. ilex* is higher than that of other *Quercus* spp. such as *Q. robur* (0.07 Mb; Plomión et al., 2018), *Q. suber* (0.08 Mb; Ramos et al., 2018), *Q. acutissima* (1.44 Mb; Liu et al., 2022), *Q. lobata* (1.90 Mb; Sork et al., 2022), and *Quercus mongolica* (2.64 Mb; Ai et al., 2022). BUSCO, in the genome assembling, predicted gene models exhibiting 94.80% of complete and single-copy genes, with a low percentage of duplicated (4.20%), fragmented (0.20%), and missing (0.80%) genes (Supplementary Table S2), indicating that the assembled genome has a high quality and is nearly complete. Similar results were observed in *Q. lobata* (94%) (Sork et al., 2016), *Q. suber* (95%) (Ramos et al., 2018), and *Quercus gilva* (93.5%) (Zhou et al., 2022). A total of 493 contigs, accounting for 835 Mbp (99% of the total assembly), were found to have some correspondence on *Q. lobata* chromosomes. The relative size of each chromosome varied from 43.58 Mbp (chromosome 12) to 106.09 Mbp (chromosome 2) (Supplementary Table S3). The number of *Q. ilex* contigs assigned to each chromosome ranged from 20 (chromosome 12) to 71 (chromosome 4) (Supplementary Table S3). Less than 1% of the sequences (37 contigs, size: 6.69 Mbp) failed to be assigned, and they were merged and placed in chromosome 0 (chr 0). Arbitrary gaps of 100 bp were added between consecutive contigs (517 gap sequences, 51.7 Kbp).

**TABLE 1:** Comparison of summary statistics for genome assembly among *Quercus* species.

Species	Assembled genome size (Mpb)	Contig N50 (Mbp)	Repetitive element (% of genome)	Protein-coding gene (*n*)	Reference
*Q. ilex*	842.2	3.30	53.3	39,443	This study
*Q. robur*	814.3	16.00	53.3	25,808	Plomión et al. (2016); Plomión et al. (2018)
*Q. lobata*	845.9	0.97	54.4	38,373	Sork et al. (2016), Sork et al. (2022)
*Q. suber*	953.5	0.08	12.0	33,658	Ramos et al. (2018)
*Q. variabilis*	787.0	64.90	67.6	32,466	Han et al. (2022)
*Q. gilva*	889.8	28.30	57.6	36,442	Zhou et al. (2022)
*Q. mongolica*	809.8	2.40	53.8	36,553	Ai et al. (2022)
*Q. acutissima*	957.1	1.20	57.1	29,889	Liu et al. (2022)
*Q. dentata*	893.5	4.21	47.0	31,584	Wang et al. (2023)

### Repetitive sequences

Repetitive sequences in the *Q. ilex* genome assembly accounted for 53.3% (448.68 Mb) of the total genome length (Supplementary Table S4). LTR Gypsy and Copia were the most abundant repeat types (14.37% and 11.58%, respectively). The proportion of repetitive elements in the *Q. ilex* genome was similar to that of repetitive elements in other *Quercus* genomes with 51.78% in *Q. mongolica* (Ai et al., 2022), 53.3% in *Q. robur* (Plomion et al., 2018), 53.8% in *Q. mongolica* (Ai et al., 2022), and 54.4% in *Q. lobata* (Sork et al., 2022) (Table 1). However, this proportion was higher in the *Q. gilva* (57.57%) (Zhou et al., 2022), *Quercus variabilis* (67.7%) (Han et al., 2022), and *Quercus acutissima* (57.13%) genomes (Liu et al., 2022).

### Genome prediction and functional annotation

The final gene set contained 39,443 protein-coding genes (Supplementary Tables S5, S6) and 759 tRNA genes (Supplementary Table S7), which is similar to other *Quercus* spp. (Table 1). The annotated genome showed a completeness >98% with a high degree of single-copy genes (>95%) and a low percentage of duplication (3.1%) (Supplementary Table S2). Of the total number of 39,443 genes, 35,258 (89.4%), 29,507 (74.8%), and 9,555 (24.2%) were identified with functional descriptions, GO terms, and path maps (KEGG), respectively (Supplementary Table S5). The remaining 4,185 (10.6%) genes were described without annotation. The GO representation of the whole gene set according to their biological processes, cellular components, and molecular functions is summarized in Supplementary Figure S4. Within the biological processes, the most abundantly assigned genes were involved in protein phosphorylation, the oxidation–reduction process, and defense response. In the cellular component category, the genes fell mainly into the categories of integral components of the membrane, nucleus, and plasma membrane. In the case of the molecular functions, many genes were associated with ATP binding, metal ion binding, and protein binding.

### Ortholog identification

**FIGURE 1:**
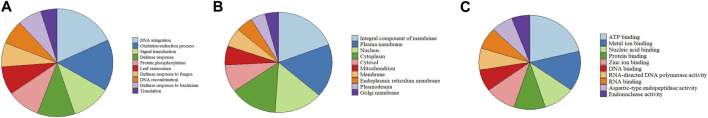
GO classification of *Q. ilex*-specific genes. Distribution of GOs associated with the *Q. ilex* genes represented in the three main GO categories: biological processes **(A)**, molecular functions **(B)**, and cellular components **(C)**. The first 10 GO groups are shown, and the remaining GO groups are shown in Supplementary Table S9.

We identified orthologous gene families by comparing the proteomes of *Q. ilex*, which were predicted in our project, with those of seven other flowering plant species, including *Q. suber*, *Q. lobata*, *Q. robur*, *J. regia*, *P. trichocarpa*, and *A. thaliana* (Supplementary Table S8). OrthoFinder assigned 35,861 orthogroups and 273,514 genes in orthogroups (out of 305,119 genes, 89.6%), with 31,605 genes remaining unassigned (10.4%). A total of 11,149 orthogroups were found to be common to all the species, and 11,807 were found to be species specific. Out of the 273,514 genes, 1,206 were found to be unique to the *Q. ilex* genome (Supplementary Table S9). The GO representation of the *Q. ilex* specific gene set according to their biological processes, cellular components, and molecular functions is shown in Supplementary Table S9. Within the biological processes, the top three associated categories were DNA integration, the oxidation–reduction process, and signal transduction (Figure 1A). In the cellular component category, the majority of genes were classified in terms of integral components of the membrane, plasma membrane, and nucleus (Figure 1B). In the case of the molecular functions, many genes were associated with ATP binding, metal ion binding, and protein binding (Figure 1C).

### Genome synteny and whole-genome duplications

**FIGURE 2:**
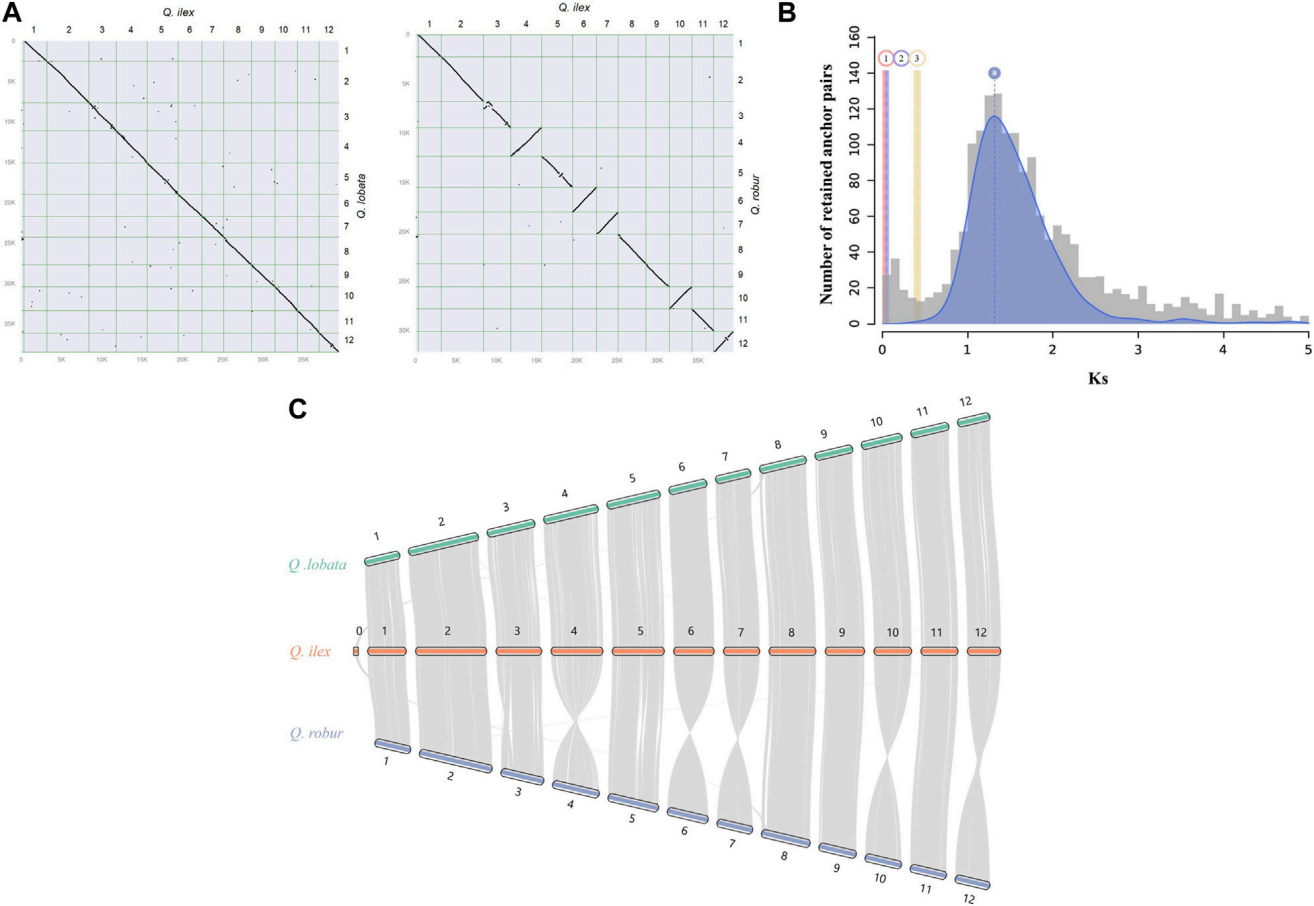
Genome synteny between *Q. ilex* and other species evaluated in this work. **(A)** Synteny dot plot between *Q. ilex* and *Q. lobata* (left) and between *Q. ilex* and *Q. robur* (right), with syntenic blocks being signified by diagonal lines sloping down and by inverted syntenic blocks being signified by diagonal lines sloping up. **(B)** WGD signatures in *Q. ilex*. The histogram shows the substitution-rate-adjusted mixed paralog -ortholog Ks plot for *Q. ilex* (paralogs, gray) and *Q. ilex* vs. *Q. lobata* (orthologs, red), *Q. ilex* vs. *Q. suber* (orthologs, violet), and *Q. ilex* vs. *J. regia* (ortholog outgroup, yellow). **(C)** Chromosome-level syntenic comparisons between Q. ilex (orange) and *Q. robur* (blue) and between *Q. ilex* (orange) and *Q. lobata* (green). Gray lines represent gene pairs in syntenic blocks.

In the synteny analysis between *Q. ilex* and *Q. lobata* and between *Q. ilex* and *Q. robur*, a conserved collinearity among genomic blocks and a clear one-to-one relationship of the 12 chromosomes were observed (Figures 2A, B). This analysis allowed us to infer that all misassembled scaffolds in our assembly (placed in chr0) likely belong to chromosome 8 (Figure 2B) (Sork et al., 2022). Liu et al. (2022) and Han et al. (2022) reported a largely conserved gene synteny between *Q. variabilis* and *Q. acutissima* and between *Q. variabilis* and *Q. lobata* and *Q. robur*, respectively. Therefore, this suggests little rearrangement of chromosomes of the genus *Quercus*, indicating conserved evolution in Fagaceae.

The potential WGD events in the evolutionary history of *Q. ilex* were studied by the distribution of the Ks between homologous gene pairs derived from *Q. ilex*, *Q. lobata*, *Q. suber*, and *J. regia*. One main peak was observed in the *Q. ilex* genome based on the abundancy of Ks value (a Ks value of 1.31), indicating a single major WGD event (Figure 2C) (Han et al., 2022; Zhou et al., 2022). The low Ks values between *Q. ilex* and *Q. lobata* (0.04–0.06 Ks units) and between *Q. ilex* and *Q. suber* (0.02–0.04 Ks units) indicated little divergence between these three species, supported by the higher divergence with *J. regia* (0.41–0.42 Ks units) (Figure 2C).

### Expanded and contracted gene families

**FIGURE 3:**
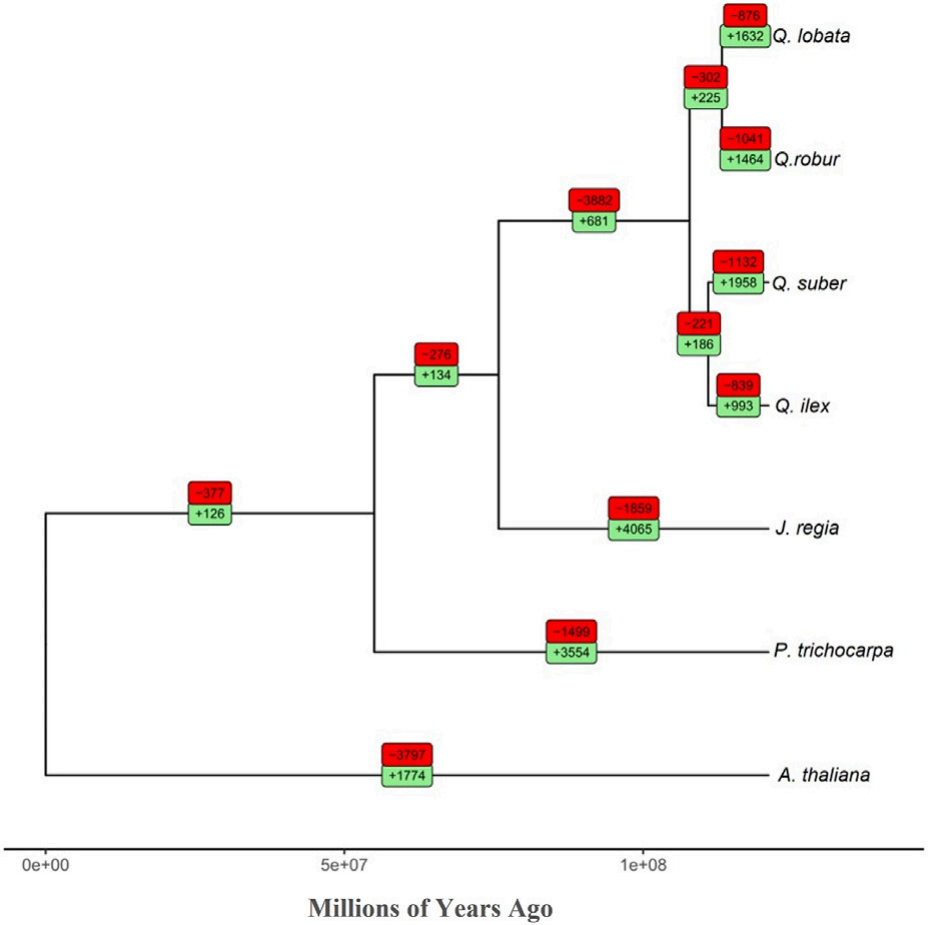
Phylogenomic tree (total age: 121 MYA) of the seven species analyzed in this study. The red and green boxes show the number of contracted and expanded gene families per branch, respectively.

We analyzed gene family expansions and contractions in the seven species used in the OrthoFinder analysis. We found that the ratio between contracted versus expanded gene families in *Q. ilex* (839/993) was much lower than in the other *Quercus* spp. included in our analysis (*Q. suber*, 1132/1958; *Q. robur*, 1041/1464; and *Q. lobata*, 876/1632) (Figure 3). In addition, this ratio was also lower than that in *Q. acutissima* (2390/3897) (Liu et al., 2022). *Q. ilex* exhibited a notable enrichment of expanded genes involved in “glutathione metabolism,” “plant hormone signal transduction,” “oxidative phosphorylation,” “peptidases and inhibitors,” “phenylpropanoid biosynthesis,” and “sesquiterpenoid and triterpenoid biosynthesis,” while contracted genes were enriched in “glycosyltransferases” and “ribosome” (Supplementary Table S10).

### Chloroplast genome assembly and annotation

**FIGURE 4:**
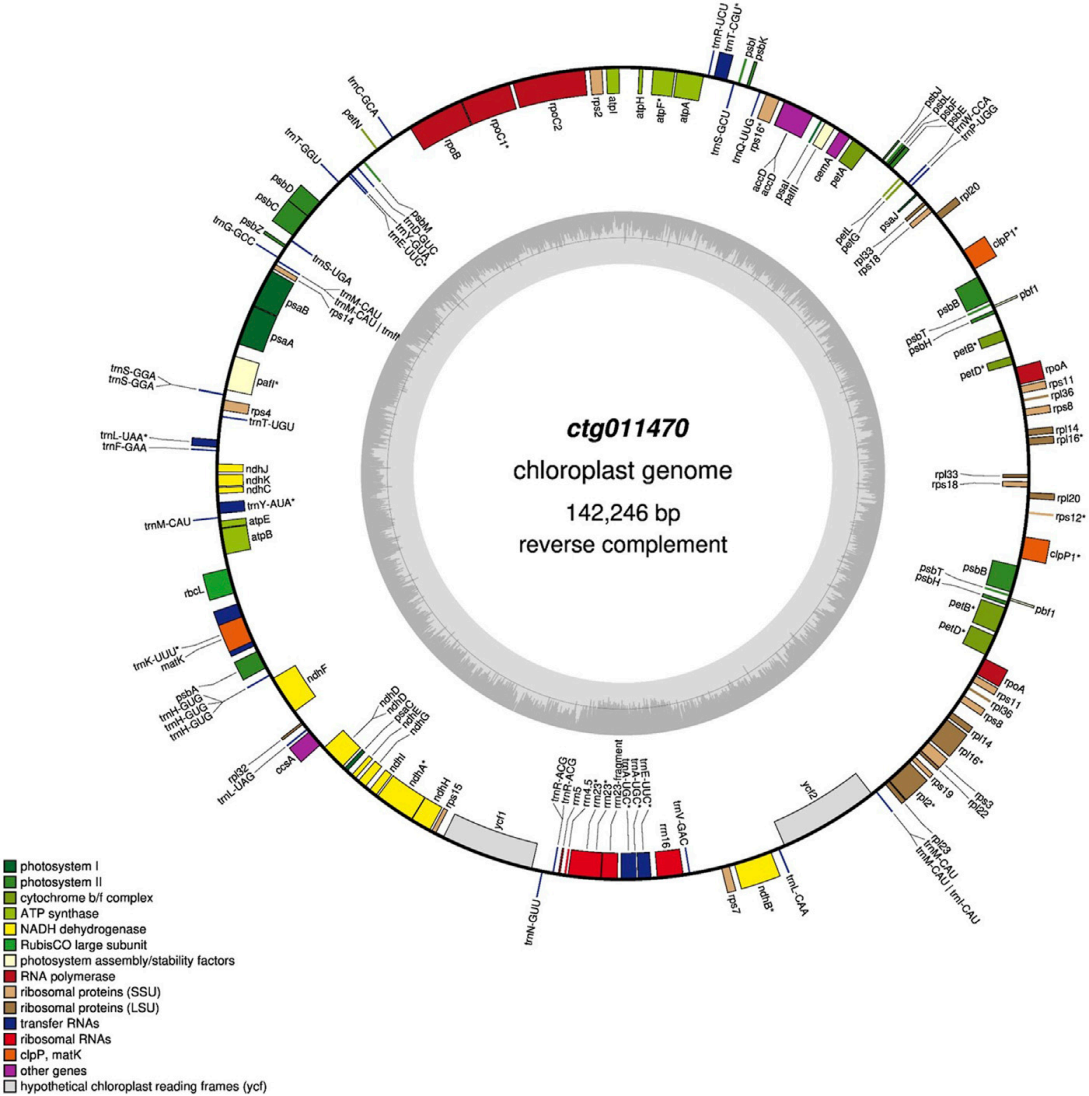
Gene map of the *Q. ilex* chloroplast genome. Genes shown outside and inside the outer circle are transcribed clockwise and counterclockwise, respectively. The darker and lighter gray areas in the inner circle indicate the GC and AT content, respectively.

One contig (ctg011470, ∼140 Kbp) showed high homology with *Medicago sativa* chloroplast sequences, and five contigs (ctg008800, ctg009160, ctg009300, ctg009310, and ctg011650) showed homology with mitochondrial sequences of different plant species (*Q. robur*, *Q. acutissima*, and *Lithocarpus litseifolius*). The final chloroplast assembly was 142.3 Kbp in size with a GC content of 33.5%. We annotated 149 genes of which 92 were protein-coding genes, 51 were rRNAs, and six were rRNAs (Figure 4). A comparison of features across 28 chloroplast genomes of the genus *Quercus* is included in Supplementary Table S11. All the chloroplast genomes exhibited a consistent size, except for *Q. ilex*, which displayed a smaller genome size. The number of genes ranged from 113 (e.g., *Quercus wutaishanica*, *Quercus rubra*, and *Quercus macrocarpa*, among others) to 140 (*Q. ilex*). In terms of GC content, *Q. ilex* exhibited the lowest value, while *Q. lobata* displayed the highest.

## Conclusion

We generated the first draft of the *Q. ilex* genome with a high level of completeness and continuity. The final assembly is 842.2 Mbp which is similar to other *Quercus* species. We found no strong evidence of chromosome fusion or species-specific WGD events, and there was high synteny between *Q. ilex* chromosomes and those of *Q. lobata* and *Q. robur*. Gene evolution analysis revealed that *Q. ilex* experienced a much lower frequency of gene family expansions if compared to other *Quercus* species, although the reason behind this pattern remains unclear. This assembly could be improved in the future by the inclusion of sequencing data from other platforms such as Illumina and Oxford Nanopore. However, the available first draft of the assembled genome, together with previously published transcriptomics, proteomics, and metabolomics datasets (Guerrero-Sánchez et al., 2021; López-Hidalgo et al., 2021; San-Eufrasio et al., 2021; Maldonado-Alconada et al., 2022; Tienda-Parrilla et al., 2022), provides valuable information for future studies on comparative genomics, adaptation, hybridization, and epigenetics that provide the response to the changed environment as well as nutritional quality in acorns of *Q. ilex*.

## Data Availability

The raw sequencing data were deposited in the NCBI SRA database under the BioProject accession number PRJNA687489 for raw data and accession number SAMN17145625 JAVFWQ000000000 for the assembled genomes (nuclear + chloroplast).
