# Supplementary figures and images for: A first draft genome of holm oak (Quercus ilex subsp. ballota), the most representative species of the Mediterranean forest and the Spanish agrosylvopastoral ecosystem “dehesa”
Source: Front Mol Biosci. 2023 Oct 12;10:1242943. doi: 10.3389/fmolb.2023.1242943 (PMC10613499; doi:10.3389/fmolb.2023.1242943)

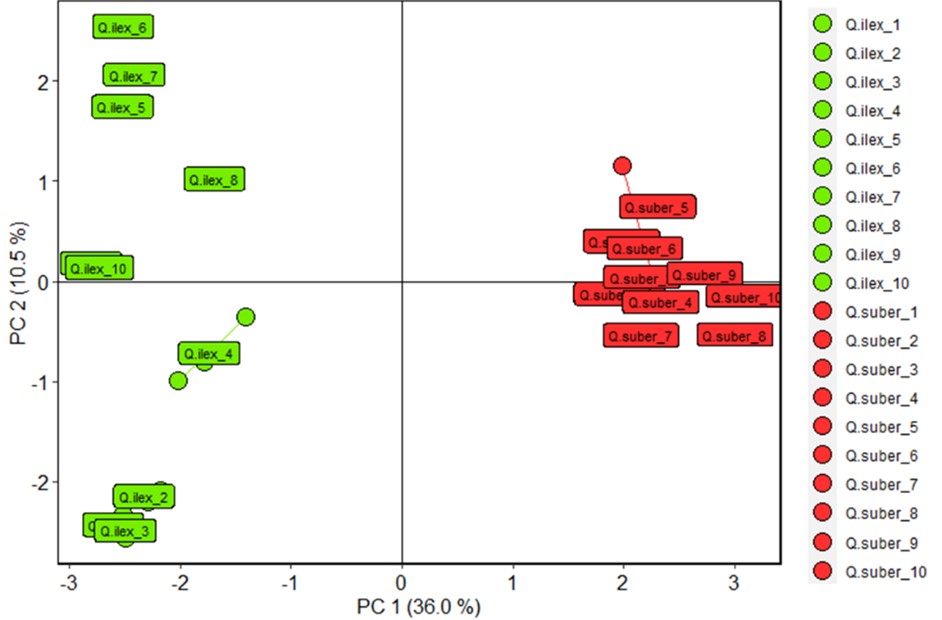

Supplement: Supplementary file 1 [file Image3.jpg]

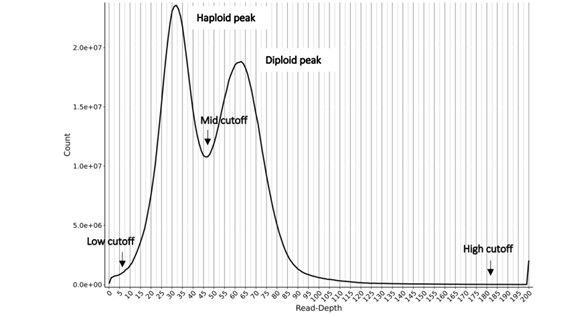

Supplement: Supplementary file 2 [file Image2.jpg]

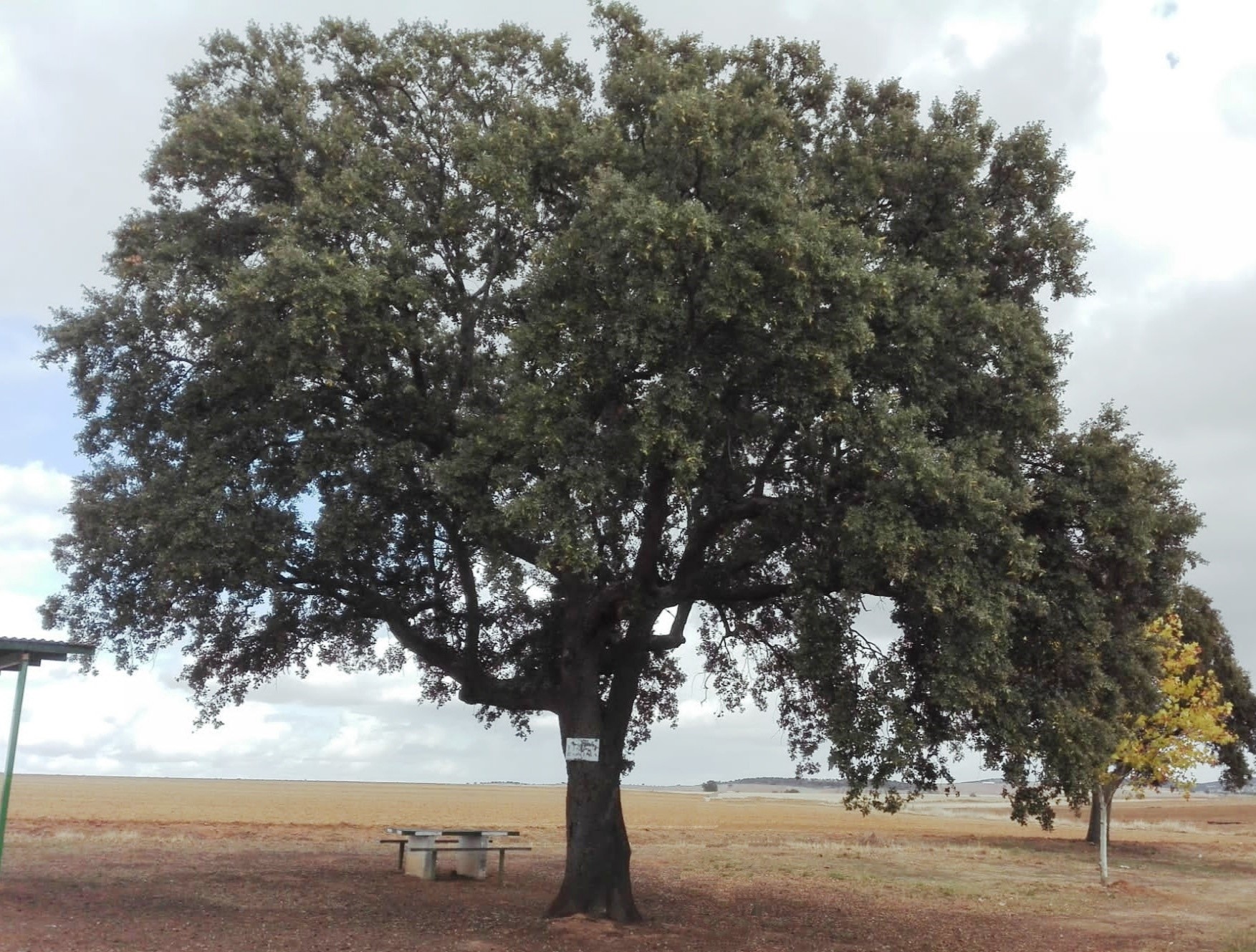

Supplement: Supplementary file 6 [file Image1.jpeg]
