# Supplementary material for: A first draft genome of holm oak (Quercus ilex subsp. ballota), the most representative species of the Mediterranean forest and the Spanish agrosylvopastoral ecosystem “dehesa”
Source: Front Mol Biosci. 2023 Oct 12;10:1242943. doi: 10.3389/fmolb.2023.1242943 (PMC10613499; doi:10.3389/fmolb.2023.1242943)
Supplement: Supplementary file 3 [file Table1.docx]

**Supplementary Table S1:** Raw and final genome assembly statistics

| **Type** | **Raw genome** | **Final genome** |
| --- | --- | --- |
| Contigs number # | 1,166 | 530 |
| Contig max size (bp) | 15,366,328 | 15,366,328 |
| Contig mean size (bp) | 858,142 | 1,589,113 |
| Contig median size (bp) | 234,957 | 846,601 |
| Contig min size (bp) | 13,813 | 17,301 |
| N50: | 2,630,716 | 3,313,408 |
| N90: | 418,639 | 883,103 |
| N95: | 205,076 | 511,677 |
| Final genome size (bp) | 1,000,594,563 | 842,230,418 |
