# Supplementary material for: A first draft genome of holm oak (Quercus ilex subsp. ballota), the most representative species of the Mediterranean forest and the Spanish agrosylvopastoral ecosystem “dehesa”
Source: Front Mol Biosci. 2023 Oct 12;10:1242943. doi: 10.3389/fmolb.2023.1242943 (PMC10613499; doi:10.3389/fmolb.2023.1242943)
Supplement: Supplementary file 4 [file Table8.docx]

**Supplementary Table S8:** Quantitative statistics of gene clustering into families.

| **Species** | **Number of genes** | **Number of genes in orthogroups** | **Number of unassigned genes** | **Percentage of genes in orthogroups** | **Percentage of unassigned genes** | **Number of orthogroups containing species** | **Percentage of orthogroups containing species** | **Number of species-specific orthogroups** | **Number of genes in species-specific orthogroups** | **Percentage of genes in species-specific orthogroups** |
| --- | --- | --- | --- | --- | --- | --- | --- | --- | --- | --- |
| *A. thaliana* | 27628 | 24779 | 2849 | 89.7 | 10.3 | 14435 | 40.3 | 984 | 4664 | 16.9 |
| *J. regia* | 40896 | 38764 | 2132 | 94.8 | 5.2 | 16399 | 45.7 | 837 | 4685 | 11.5 |
| *P. trichocarpa* | 41335 | 35828 | 5507 | 86.7 | 13.3 | 17041 | 47.5 | 1242 | 5341 | 12.9 |
| *Q. ilex* | 39389 | 37104 | 2285 | 94.2 | 5.8 | 21334 | 59.5 | 388 | 1206 | 3.1 |
| *Q. lobata* | 39366 | 37306 | 2060 | 94.8 | 5.2 | 21444 | 59.8 | 432 | 1190 | 3 |
| *Q. suber* | 79281 | 63277 | 16004 | 79.8 | 20.2 | 28964 | 80.8 | 7742 | 22419 | 28.3 |
| *Q. robur* | 37224 | 36456 | 768 | 97.9 | 2.1 | 19231 | 53.6 | 182 | 805 | 2.2 |
