# Supplementary material for: A first draft genome of holm oak (Quercus ilex subsp. ballota), the most representative species of the Mediterranean forest and the Spanish agrosylvopastoral ecosystem “dehesa”
Source: Front Mol Biosci. 2023 Oct 12;10:1242943. doi: 10.3389/fmolb.2023.1242943 (PMC10613499; doi:10.3389/fmolb.2023.1242943)
Supplement: Supplementary file 7 [file Table7.docx]

**Supplementary Table S7:** Annotation of tRNA for the *Q. ilex* assembled genome.

| Number of genes/rRNAs | 759 |
| --- | --- |
| Number of exón | 791 |
| Number of intrón | 32 |
| Number gene overlapping | 0 |
| Number of single exon gene | 727 |
| Number of single exon tRNA | 727 |
| Total gene length (bp) | 56,693 |
| Total tRNA length (bp) | 56,693 |
| Total exon length (bp) | 56,265 |
| Total intron length (bp) | 428 |
| mean gene length (bp) | 74 |
| mean tRNA length (bp) | 74 |
| mean exon length (bp) | 71 |
| mean intron in exon length (bp) | 13 |
