# Supplementary material for: A first draft genome of holm oak (Quercus ilex subsp. ballota), the most representative species of the Mediterranean forest and the Spanish agrosylvopastoral ecosystem “dehesa”
Source: Front Mol Biosci. 2023 Oct 12;10:1242943. doi: 10.3389/fmolb.2023.1242943 (PMC10613499; doi:10.3389/fmolb.2023.1242943)
Supplement: Supplementary file 9 [file Table2.docx]

**Supplementary Table S2:** BUSCO assessment of the *Q. ilex* genome assembly (Database used: Viridiplantae) and *Q. ilex* genome annotation.

| **Category** | **Genome assembling** | |
| --- | --- | --- |
|  | **Gene number** | **Percentage** |
| Complete BUSCOs (C) | 421 | 99 |
| Complete and single-copy BUSCOs (S) | 403 | 94.8 |
| Complete and duplicated BUSCOs (D) | 18 | 4.2 |
| Fragmented BUSCOs (F) | 1 | 0.2 |
| Missing BUSCOs (M) | 3 | 0.8 |
| Total BUSCO groups searched | 425 |  |
