# Supplementary material for: A first draft genome of holm oak (Quercus ilex subsp. ballota), the most representative species of the Mediterranean forest and the Spanish agrosylvopastoral ecosystem “dehesa”
Source: Front Mol Biosci. 2023 Oct 12;10:1242943. doi: 10.3389/fmolb.2023.1242943 (PMC10613499; doi:10.3389/fmolb.2023.1242943)
Supplement: Supplementary file 10 [file Table5.docx]

**Supplementary Table S5:** Gene annotation summary for the *Q. ilex* genome.

| Number of gene/mRNA | 39,443 |
| --- | --- |
| Number of mRNAs with UTR both sides | 18,255 |
| Number of mRNAs with at least one UTR | 25,862 |
| Number of CDS | 39,443 |
| Number of exon | 192,536 |
| Number of five_prime_UTR | 20,321 |
| Number of three_prime_UTR | 23,796 |
| mean exons per mRNA | 4.9 |
| mean five_prime_UTRs per mRNA | 0.5 |
| mean three_prime_utrs per mRNA | 0.6 |
| Total gene/mRNA length (bp) | 156,941,371 |
| Total CDS length (bp) | 46,744,435 |
| Total exon length (bp) | 59,868,779 |
| Total five_prime_UTR length (bp) | 4,337,145 |
| Total three_prime_UTR length (bp) | 8,787,199 |
| mean gene/mRNA length (bp) | 3,978 |
| mean CDS length (bp) | 1,185 |
| mean exon length (bp) | 310 |
| mean five_prime_UTR length (bp) | 213 |
| mean three_prime_UTR length (bp) | 369 |
| % of genome covered by gene | 18.5 |
| % of genome covered by mRNA | 18.5 |
| % of genome covered by CDS | 5.5 |
| % of genome covered by exon | 7 |
| % of genome covered by five_prime_UTR | 0.5 |
| % of genome covered by three_prime_UTR | 1 |
