# Supplementary material for: A first draft genome of holm oak (Quercus ilex subsp. ballota), the most representative species of the Mediterranean forest and the Spanish agrosylvopastoral ecosystem “dehesa”
Source: Front Mol Biosci. 2023 Oct 12;10:1242943. doi: 10.3389/fmolb.2023.1242943 (PMC10613499; doi:10.3389/fmolb.2023.1242943)
Supplement: Supplementary file 11 [file Table3.docx]

Supplementary Table S3: Number of *Q. ilex* contigs assigned to each chromosome and relative size in bp.

| **Chromosomes** | **Number of contigs assigned** | **Size in bp** |
| --- | --- | --- |
| Chromosome00 | 37 | 6693305 |
| Chromosome01 | 37 | 61376402 |
| Chromosome02 | 40 | 106094171 |
| Chromosome03 | 53 | 72685614 |
| Chromosome04 | 71 | 95986598 |
| Chromosome05 | 54 | 94247861 |
| Chromosome06 | 30 | 56476857 |
| Chromosome07 | 36 | 52929259 |
| Chromosome08 | 30 | 70239515 |
| Chromosome09 | 32 | 58459393 |
| Chromosome10 | 46 | 63539548 |
| Chromosome11 | 44 | 59970103 |
| Chromosome12 | 20 | 43583492 |
| Total | 530 | 842282118 |
