# Supplementary material for: A first draft genome of holm oak (Quercus ilex subsp. ballota), the most representative species of the Mediterranean forest and the Spanish agrosylvopastoral ecosystem “dehesa”
Source: Front Mol Biosci. 2023 Oct 12;10:1242943. doi: 10.3389/fmolb.2023.1242943 (PMC10613499; doi:10.3389/fmolb.2023.1242943)
Supplement: Supplementary file 12 [file Table4.docx]

**Supplementary Table S4:** Whole-genome TE annotation summary statistics obtained through the EDTA pipeline.

| **Class** |  | **Count** | **bp masked** | **% masked** |
| --- | --- | --- | --- | --- |
| LTR | Copia | 102,055 | 97,565,066 | 11.58% |
|  | Gypsy | 87,789 | 121,041,114 | 14.37% |
|  | Unknown | 91,052 | 53,228,573 | 6.32% |
| TIR | CACTA | 48,175 | 13,469,927 | 1.60% |
|  | Mutator | 140,582 | 47,513,167 | 5.64% |
|  | PIF_Harbinger | 56,759 | 16,009,547 | 1.90% |
|  | Tc1_Mariner | 7,879 | 1,855,567 | 0.22% |
|  | hAT | 63,303 | 22,498,218 | 2.67% |
| nonTIR | Helitron | 242,187 | 75,502,546 | 8.96% |
| Total | Interspersed | 839,781 | 448,683,725 | 53.27% |
