# Supplementary material for: A first draft genome of holm oak (Quercus ilex subsp. ballota), the most representative species of the Mediterranean forest and the Spanish agrosylvopastoral ecosystem “dehesa”
Source: Front Mol Biosci. 2023 Oct 12;10:1242943. doi: 10.3389/fmolb.2023.1242943 (PMC10613499; doi:10.3389/fmolb.2023.1242943)
Supplement: Supplementary file 13 [file Table11.docx]

**Supplementary Table S11:** Comparison of features of twenty-eight *Quercus* chloroplast genomes

| **Genome Features** | **Genome Size (bp)** | **Number of Genes** | **Number of Protein Coding Genes** | **Number of tRNA Genes** | **Number of rRNA Genes** | **GC Content (%)** | **Reference** |
| --- | --- | --- | --- | --- | --- | --- | --- |
| *Q. ilex* | 142,300 | 149 | 92 | 51 | 6 | 33.5 | In this work |
| *Q. acutissima* | 161,124 | 136 | 88 | 40 | 8 | 36.1 | Li et al., 2018 |
| *Q. aliena* | 161,150 | 134 | 86 | 40 | 8 | 36.8 | Yang et al., 2016; 2018 |
| *Q. aliena var. acutiserrata* | 161,153 | 134 | 86 | 40 | 8 | 36.8 | Yang et al., 2016, 2018; Pang et al., 2019 |
| *Q. aquifolioides* | 161,225 | 134 | 86 | 40 | 8 | 36.8 | Yang et al., 2018 |
| *Q. baronii* | 161,072 | 134 | 86 | 40 | 8 | 36.8 | Yang et al., 2016, 2018 |
| *Q. bawanglingensis* | 161,394 | 134 | 86 | 40 | 8 | 36.8 | Liu et al., 2019 |
| *Q. dentata* | 161,250 | 134 | 86 | 40 | 8 | 36.8 | Hu et al., 2019; Pang et al., 2019 |
| *Q. dolicholepsis* | 161,237 | 134 | 86 | 40 | 8 | 36.8 | Lu et al., 2016; Yang et al., 2016, 2018 |
| *Q. edithiae* | 160,988 | 128 | 87 | 30 | 8 | 38.6 | Yang et al., 2018 |
| *Q. fabri* | 161,285 | 113 | 79 | 30 | 4 | - | Pang et al., 2019 |
| *Q. glauca* | 160,798 | 134 | 86 | 40 | 8 | 36.9 | Yang et al., 2018 |
| *Q. gambelii* | 161,213 | 113 | 79 | 30 | 4 | - | Pang et al., 2019 |
| *Q. glandulifera var. brevipetiolata* | 161,224 | 113 | 79 | 30 | 4 | - | Pang et al., 2019 |
| *Q. lobata* | 161,304 | 137 | 89 | 40 | 4 | 40.0 | Alexander and Woeste 2014; Sork et al., 2016 |
| *Q. macrocarpa* | 161,236 | 113 | 79 | 30 | 4 | - | Pang et al., 2019 |
| *Q. mongolica* | 161,191 | 113 | 79 | 30 | 4 | - | Pang et al., 2019 |
| *Q. ningangensis* | 160,736 | 127 | 86 | 37 | 4 | 36.9 | Wang et al., 2021 |
| *Q. palustris* | 161,284 | 113 | 79 | 30 | 4 | - | Pang et al., 2019 |
| *Q. phillyraeoides* | 161,132 | 113 | 79 | 30 | 4 | - | Pang et al., 2019 |
| *Q. robur* ‘Fastigiata’ | 161,172 | 134 | 89 | 37 | 8 | 36.8 | Feng et al., 2020 |
| *Q. rubra* | 161,366 | 113 | 79 | 30 | 4 | 36.8 | Yang et al., 2018; Pang et al., 2019 |
| *Q. serrata* | 161,146 | 113 | 79 | 30 | 4 | - | Pang et al., 2019 |
| *Q. spinosa* | 161,156 | 134 | 86 | 40 | 8 | 36.8 | Yang et al., 2018 |
| *Q. stellata* | 161,214 | 113 | 79 | 30 | 4 | - | Pang et al., 2019 |
| *Q. tarokoensi*s | 161,355 | 134 | 86 | 40 | 8 | 36.9 | Yang et al., 2018 |
| *Q. variabilis* | 161,077 | 134 | 86 | 40 | 8 | 36.8 | Yang et al., 2016, 2018 |
| *Q. wutaishanica* | 161,211 | 113 | 79 | 30 | 4 | - | Pang et al., 2019 |
